# Secular trends in gabapentinoid dispensing by compensated workers with low back pain: a retrospective cohort study

**DOI:** 10.1136/oemed-2023-109369

**Published:** 2024-05-23

**Authors:** Stephanie Mathieson, Alex Collie, Christopher G Maher, Christina Abdel Shaheed, Ting Xia, Stephen Gilbert, Giovanni E Ferreira, Michael F Di Donato

**Affiliations:** 1 Sydney Musculoskeletal Health, The University of Sydney, Sydney, New South Wales, Australia; 2 School of Public Health and Preventive Medicine, Monash University, Melbourne, Victoria, Australia; 3 Institute for Musculoskeletal Health, The University of Sydney, Sydney, New South Wales, Australia; 4 Monash Addiction Research Centre, Eastern Health Clinical School, Monash University, Melbourne, Victoria, Australia

**Keywords:** Back Pain, Veterans, Health services research

## Abstract

**Objectives:**

The increase in gabapentinoid prescribing is paralleling the increase in serious harms. To describe the low back pain workers compensation population whose management included a gabapentinoid between 2010 and 2017, and determine secular trends in, and factors associated with gabapentinoid use.

**Methods:**

We analysed claim-level and service-level data from the Victorian workers’ compensation programme between 1 January 2010 and 31 December 2017 for workers with an accepted claim for a low back pain injury and who had programme-funded gabapentinoid dispensing. Secular trends were calculated as a proportion of gabapentinoid dispensings per year. Poisson, negative binomial and Cox hazards models were used to examine changes over time in incidence and time to first dispensing.

**Results:**

Of the 17 689 low back pain claimants, one in seven (14.7%) were dispensed at least one gabapentinoid during the first 2 years (n=2608). The proportion of workers who were dispensed a gabapentinoid significantly increased over time (7.9% in 2010 to 18.7% in 2017), despite a reduction in the number of claimants dispensed pain-related medicines. Gabapentinoid dispensing was significantly associated with an opioid analgesic or anti-depressant dispensing claim, but not claimant-level characteristics. The time to first gabapentinoid dispensing significantly decreased over time from 311.9 days (SD 200.7) in 2010 to 148.2 days (SD 183.1) in 2017.

**Conclusions:**

The proportion of claimants dispensed a gabapentinoid more than doubled in the period 2010–2017; and the time to first dispensing halved during this period.

**WHAT IS ALREADY KNOWN ON THIS TOPIC**

- The increase in gabapentinoid prescribing is paralleling the increase in serious harms such as misuse, abuse and death, but it is unclear on gabapentinoid dispensing trends in people with worker’s compensation claims who have a primary issue of low back pain.

**WHAT THIS STUDY ADDS**

- The proportion of workers dispensed a gabapentinoid significantly increased over time and the time to first dispensing shortened.
- One in seven low back pain claimants were dispensed a gabapentinoid at least once during the first 2 years of their claim.
- Gabapentinoid dispensing was significantly associated with an opioid analgesic or anti-depressant claim.

**HOW THIS STUDY MIGHT AFFECT RESEARCH, PRACTICE OR POLICY**

- Although the proportion of analgesic medicines claimed by people with low back pain-related worker’s compensation claims decreased over time, gabapentinoid use increased.

## Introduction

Low back pain is the leading cause of disability worldwide.^1^ Of the >500 million people estimated to experience back pain globally, the prevalence is greater in women than men and prevalence increases with age.^1^ Back pain is commonly experienced in the working age group,^2^ and survey data indicates that one in five workers with work-related low back pain seek workers’ compensation for their back injury, and claim filing is more frequent among workers in the 45–64 years age group.^3^ Data from Australian workers’ compensation programmes indicates that the median time off work is 9 weeks in those people with a primary compensation claim related to the low back.^4^

The management of low back pain commonly includes pharmacological management. Some clinical practice guidelines for managing low back pain now recommend avoiding some medicines, such as opioid analgesics and gabapentinoids (pregabalin, gabapentin),^5^ as the benefit often does not outweigh the harms. In people with work-related low back compensation claims,^6^ opioid analgesics have been found to lead to prolonged work disability with an increased daily dose,^7^ compared with other medicines like non-steroidal anti-inflammatory drugs^8^ and are associated with increased opioid-related deaths.^9^ The increasing incidence of gabapentinoid-related harms have been documented in the literature, such as abuse, misuse, dependence or overdose.^10 11^ However, the extent of gabapentinoid-related harms in work-related low back compensation claims is not well known.

Gabapentinoids are anti-epileptic drugs that are approved to treat a small number of neuropathic pain conditions, such as post-herpetic neuralgia.^12^ But in recent times, there has been a shift in increased ‘off-label’ prescribing (ie, for non-approved conditions) partially in response to clinicians seeking a non-opioid alternative following increased awareness of opioid harm.^13^ In Australia, low back pain is a key driver of off-label pregabalin prescribing,^14^ and there have been increases in gabapentinoid prescribing to patients with low back pain in primary care.^15^ The use of gabapentinoids for back pain can be associated with providing low-value care, that is, when the probable benefits do not exceed the potential harms.^16^ For example, pregabalin provides no greater pain relief than placebo in patients with sciatica (a severe form of back pain and leg pain) but with an increased rate of adverse events.^17^

Although gabapentinoid prescribing has increased in Australia^14 15^ and internationally^18 19^ over the last decade, it is unclear if similar prescribing trends have occurred in workers’ compensation populations. The extent to which gabapentinoids are prescribed in workers’ compensation cohort is infrequently reported. Analysis of North American jurisdiction (Louisiana) workers’ compensation claims revealed a doubling in gabapentin claims between 2008 and 2018, and an 80% decrease in pregabalin reimbursement claims during the same time. However, these trends are for a single geographical location of private insurance claims, and the extent these trends are associated with low back pain injuries is unknown.^20^ Understanding the secular prescribing trends in workers can give insight into whether workers’ compensation claimants receive gabapentinoids to manage their back pain. Therefore, this study aimed to examine gabapentinoid dispensing between 2010 and the end of 2019 in a low back pain workers’ compensation population. A second aim was to determine factors associated with gabapentinoid dispensing.

## Methods

### Database

This study analysed retrospective cohort data from the compensation database of the workers’ compensation regulator in the state of Victoria, Australia, the second most populous state. The Victorian workers’ compensation programme covers approximately 85% of 3.2 million Victorian workers in 2017. A standard claim is recorded in the database once 10 days have been lost from work, or a threshold of healthcare expenditure has been reached (~$A700 in the 2018/2019 financial year). The healthcare expenditure includes reimbursement to the payee for reasonable costs related to the work-related injury or illness.

### Sample

Workers aged 15–80 years with accepted workers’ compensation time-loss claims for low back pain received by the insurer between the 1 January 2010 and 31 December 2017 were included. Time loss claims were those with at least 1 day of workers’ compensation-funded income replacement. Low back pain was defined using the database’s coding system, Vcode (online supplemental appendix 1). Claimant details included variables related to the claim (claim filing and approval date, date of injury, details of injury, details of services provided per claimant, such as date, cost, for physician consultations, imaging referrals); and claimant (worker) variables (age group (15–24 years, 25–34 years, 35–44 years, 45–54 years, 55–64 years, >65 years), gender (male, female), employer size (small (<$A1 million annual turnover), medium ($A1–20 million annual turnover), large (>$A20 million annual turnover, government)), employment type (full-time (≥35 hours/week), part-time, casual, other), Australian Standard Classification of Occupations occupation category (clerical, professional, labourer, manager, tradesperson)^21^). Index of Relative Socio-economic Advantage and Disadvantage socioeconomic status (in quintiles)^22^ and Accessibility/Remoteness Index of Australia remoteness (major city, inner regional, outer regional and remote^23^)) defined by a workers’ postcode.

10.1136/oemed-2023-109369.supp1 Supplementary data

#### Medicines data set

Medication variables available included drug name, drug Anatomical Therapeutic Chemical (ATC) code, drug strength, pack size dispensed, service cost, year of claim, claimants’ approval date. Gabapentinoids were either pregabalin or gabapentin (ATC code N02BF). Gabapentinoid dispensing were reimbursed for 1-month prior to the claim approval date to 24 months post claim date, the last available date in the data set. Medicines considered for pain management included ATC codes of M01, M02, M03, N01, N02, N03, N05, N06.

### Data management

Following an agreement with the regulator of the compensation system, WorkSafe Victoria, data were received and analysed using established secure protocols. Researchers conducting the analyses were granted access to the data stored on Monash University’s virtual server platform and analyses conducted within Monash’s Secure eResearch Platform. A summary of high-level collated analyses was exported from the environment.

### Data synthesis

Claimant variables are described per data category except for socioeconomic status, which were grouped into categories of most disadvantaged (quintile 1), middle three quintiles and most advantaged (quintile 5). The number of days to a first dispensing claim of a gabapentinoid was determined from the insurer received date for the claim to the date associated with the first gabapentinoid dispensing. A new episode of gabapentinoid use was considered if there were more than 60 days between gabapentinoid dispensing. Where present, missing data are reported per variable (n/N (%)). There were no missing data related to medicine variables.

### Analyses

The characteristics of the claimant population were described with proportions (n/N (%)), means and SD or median and IQR as appropriate. The proportion of claimants who claimed a gabapentinoid was determined per year. A Poisson model examined gabapentinoid dispensing over time, adjusting for all available covariates with a log link and offset by the log of the number of total claims, reported as prevalence ratio with 95% CIs. A negative binomial model determined associations with the number of gabapentinoids dispensed per claimant adjusted for all available covariates and reported as an incidence rate ratio with 95% CI. A Cox proportional hazards model determined associations with the time to first gabapentinoid dispense per claimant adjusted for all available covariates and reported as HR with 95% CI. Statistical analyses were conducted in R V.4.2.2 (Vienna, Austria).

## Results

The final sample included 17 689 low back pain claimants. The sample is described in table 1. Of the low back claimants, there were 159 654 dispensing claims for any type of medicine considered for pain management. Analgesics (N02) were the most common medication dispensed (n=97 598, 61.1%).

**Table 1:** Description of low back pain claimants (n=17 689) and those dispensed at least one gabapentinoid between 2010 and 2017

Characteristic	Low back pain claimants	Low back pain claimants dispensed at least one gabapentinoid		
	N (%)	N (% back pain claims)	Prevalence ratio (95% CI)	P value
Total	17 689 (100)	2608 (100)		
Sex				
Female	6301	876 (13.9)	0.96 (0.89 to 1.04)	0.474
Male	11 388	1732 (15.2)	1.00 (ref)	–
Age group				
15–24 years	1514 (8.6)	105 (6.9)	0.77 (0.65 to 0.91)	0.016
25–34 years	3846 (21.7)	520 (13.5)	0.94 (0.86 to 1.02)	0.260
35–44 years	4492 (25.4)	777 (17.3)	1.02 0.94 to 1.09)	0.771
45–54 years	4724 (26.7)	761 (16.1)	1.00 (ref)	–
55–64 years	2878 (16.3)	416 (14.5)	0.93 (0.85 to 1.02)	0.247
65 or more years	235 (1.3)	29 (12.3)	0.94 (0.71 to 1.25)	0.747
Employer size				
Small	4137 (23.4)	672 (16.2)	1.08 (1.00 to 1.16)	0.148
Medium	7161 (40.5)	1058 (14.8)	1.00 (ref)	–
Large	4805 (27.2)	706 (14.7)	1.02 (0.95 to 1.10)	0.679
Government	679 (3.8)	99 (14.6)	1.06 (0.90 to 1.25)	0.592
Missing	907 (5.1)	73 (8.0)	–	–
Employment type			1.02 (0.85 to 1.22)	0.918
Casual	262	47 (17.9)	1.00 (ref)	–
Full-time employee	12 137	1869 (15.4)	0.96 (0.87 to 1.06)	0.532
Part-time employee	3071	417 (13.6)	0.96 (0.88 to 1.06)	0.576
Others	2219	275 (12.4)		
Occupation (ASCO)				
Advanced clerical and service workers	160 (0.9)	14 (8.8)	0.78 (0.52 to 1.19)	0.369
Associate professionals	1778 (10.0)	215 (12.1)	0.98 (0.86 to 1.13)	0.852
Elementary clerical, sales and service workers	714 (4.0)	115 (16.1)	1.12 (0.97 to 1.29)	0.283
Intermediate clerical, sales and service workers	2259 (12.8)	304 (13.5)	0.96 (0.86 to 1.07)	0.579
Intermediate production and transport workers	3476 (19.7)	564 (16.2)	1.02 (0.94 to 1.12)	0.689
Labourers and related workers	3668 (20.7)	553 (15.1)	1.00 (ref)	–
Managers and administrators	501 (2.8)	96 (19.2)	1.07 (0.90 to 1.26)	0.569
Professionals	2008 (11.4)	290 (14.4)	1.07 (0.95 to 1.21)	0.407
Tradespersons and related workers	3125 (17.7)	457 (14.6)	1.06 (0.97 to 1.16)	0.377
Socioeconomic status (IRSAD)				
Most advantaged	3046 (17.2)	354 (11.6)	0.96 (0.88 to 1.05)	0.535
Middle three quintiles	11 742 (66.4)	1747 (14.9)	1.00 (ref)	–
Most disadvantaged	2868 (16.2)	498 (17.4)	0.99 (0.92 to 1.07)	0.885
Missing	33 (0.2)	9 (27.3)	–	–
Remoteness (ARIA)				
Major cities	12 830 (72.6)	1841 (14.3)	1.00 (ref)	–
Inner regional	4022 (22.7)	647 (16.1)	1.08 (1.01 to 1.15)	0.122
Outer regional and remote	818 (4.6)	115 (14.1)	0.94 (0.81 to 1.09)	0.562
Missing	19 (0.1)	5 (26.3)	–	–
Dispensed opioid analgesics (N02)				
Dispensed an opioid analgesic(s)	5541 (31.3)	2365 (42.7)	14.07 (12.18 to 16.24)	<0.001
No opioid analgesics	12 148 (68.7)	243 (2.0)	1.00 (ref)	
Dispensed anti-depressants (N06A)				
Dispensed an anti-depressant(s)	2476 (14.0)	1466 (59.2)	2.24 (2.09 to 2.39)	<0.001
No anti-depressants	15 213 (86.0)	1142 (7.5)	1.00 (ref)	
Received any pain-related medicine*		–	–	–
Dispensed any pain medicine	6344 (35.9)	2519 (39.7)	–	–
No other pain medicine (ie, excluding N02BF)	11 345 (64.1)	89 (0.8)	–	–

*Medicine used for pain management included Anatomical Therapeutic Chemical (ATC) codes of M01 (anti-inflammatory and antirheumatic products, non-steroids), M02 (topical products for joint and muscular pain), M03 (muscle relaxants), N01 (anaesthetics), N02 (analgesics), N03 (anti-epileptics), N05 (psycholeptics), N06 (psychoanaleptics). ATC code of N06A are anti-depressants, and N02BF are gabapentinoids.

ARIA, Accessibility/Remoteness Index of Australia; ASCO, Australian Standard Classification of Occupations; IRSAD, Index of Relative Socio-economic Advantage and Disadvantage.

**Figure 1:**
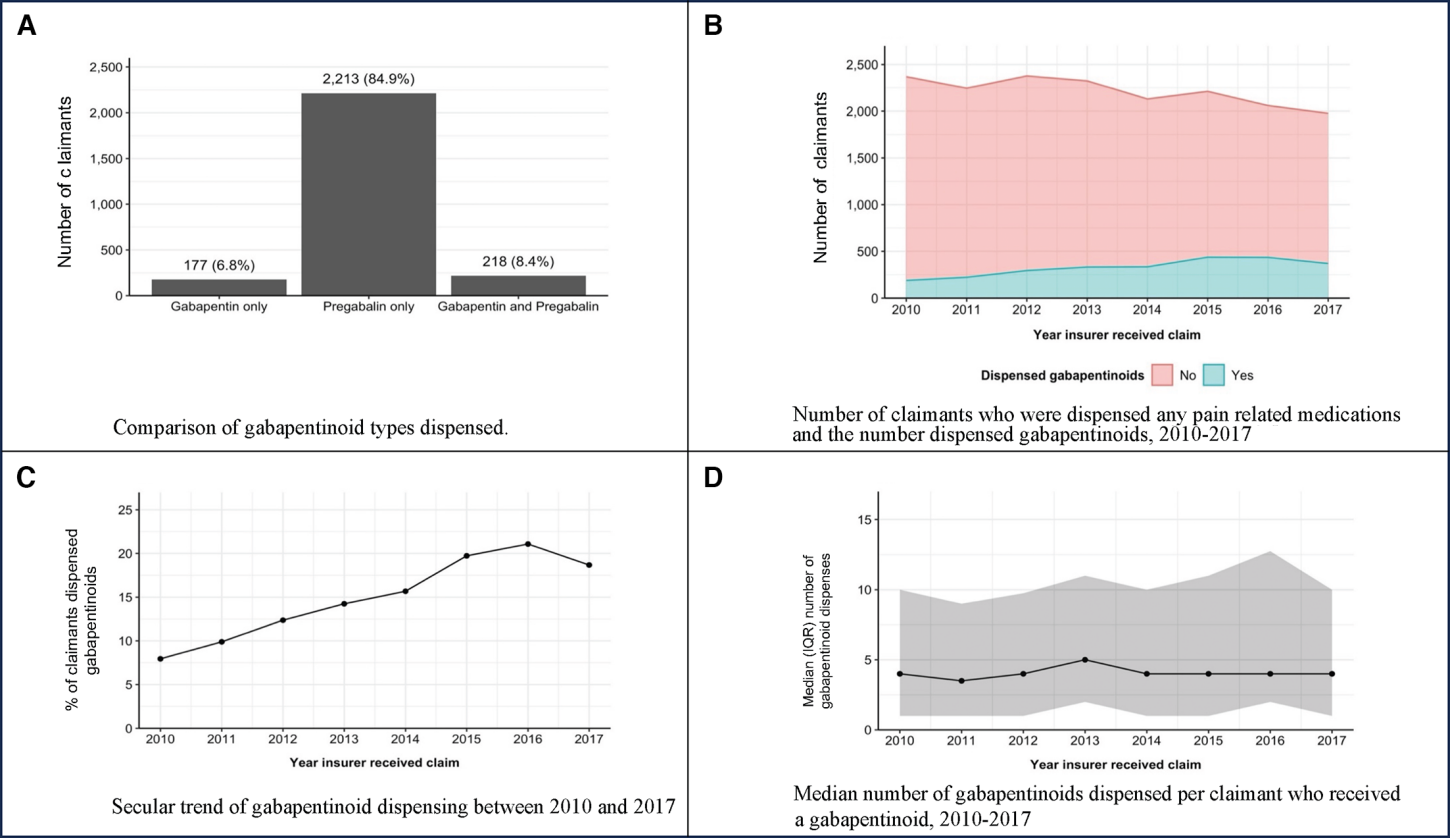
Characteristics of gabapentinoid dispensing. (A) Comparison of gabapentinoid types dispensed. (B) Yearly gabapentinoid dispensing out of all pain-related medicines. (C) Secular trend of gabapentinoid dispensing between 2010 and 2017. (D) Number of gabapentinoid dispensed per claimant over time.

One in seven low back claimants were dispensed a gabapentinoid at least once during the first 2 years of their claim (n=2608, 14.7%) (table 1). Pregabalin accounted for 84.9% of claimants dispensed a gabapentinoid (n=2608) (figure 1A). Gabapentin dispensing was small (n=177) and stable over time. Concomitant dispensing of both pregabalin and gabapentin at any time point was infrequent (n=218; 8.4%) (figure 1A). The proportion of claimants dispensed a gabapentinoid increased from 7.9% in 2010 to 21.7% in 2016 (p=0.041) (figure 1C). Yearly values are presented in online supplemental appendix 2. Gabapentinoid dispensing was significantly associated with opioid analgesic and anti-depressant dispensing, but not any claimant-level characteristics (table 1).

Gabapentinoid dispensing increased over time despite reducing the number of low back pain claimants being dispensed pain-related medicines (figure 1B). The majority of workers who were dispensed gabapentinoids were also dispensed an opioid analgesics(s) during their claim (90.7%, n=2365). Of those 2365 workers, 67.3% were dispensed opioids prior to gabapentinoids (online supplemental appendix 4). Online supplemental appendix 4 details the proportion of workers who dispensed other pain medicines before or after their gabapentinoid dispensing.

**Figure 2:**
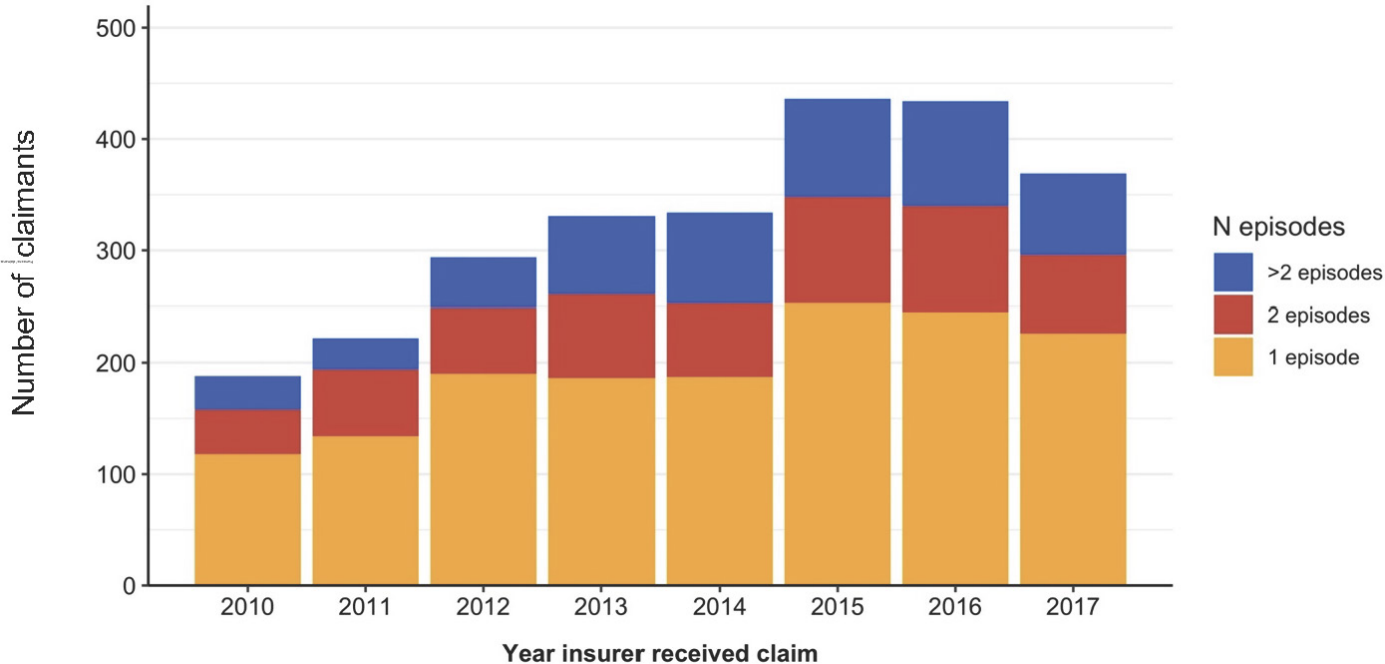
Number of episodes per claimant over time. A new episode of gabapentinoid use was considered if there was more than 60 days between gabapentinoid dispensing.

Most claimants had one episode of gabapentinoid dispensings (figure 2, online supplemental appendix 3). The mean number of gabapentinoid dispensing per claimant was 7.4 (SD 8.2). A lower number of dispenses per claimant was associated with those who were older (65 years or older) (p=0.034) compared with those 45–54 years; tradespersons occupation compared with labourers (p=0.270); those in most advantaged economic categories compared with the middle quintiles (p=0.003); and those living within inner regional areas compared with claimants living in major cities (p=0.078). Other claimant characteristics were not statistically significant.

**Figure 3:**
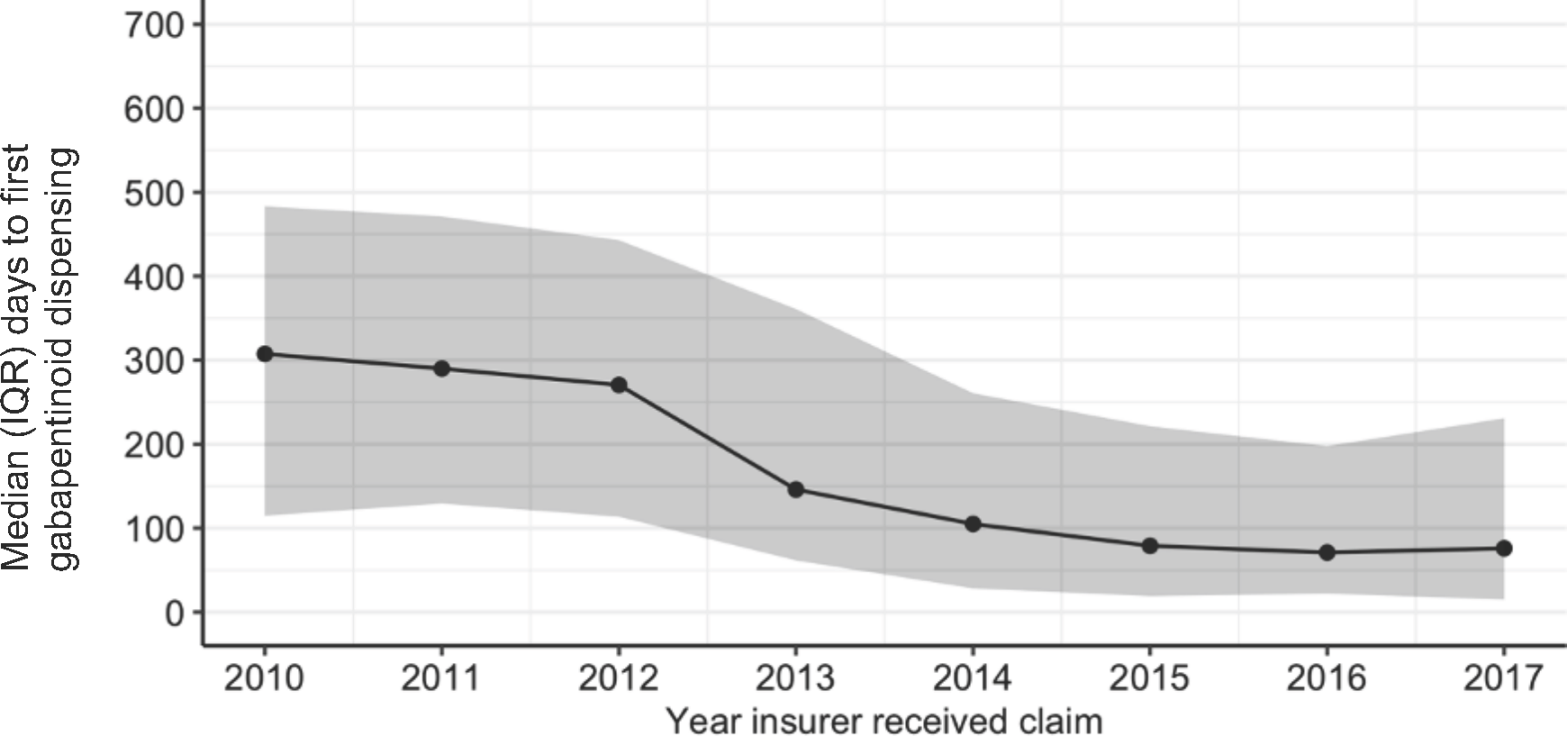
Median number of days to first gabapentinoid dispensing over time.

The time to first gabapentinoid dispensing significantly decreased over time (figure 3, online supplemental appendix 2). The mean number of days to first dispensing was 311.9 days (SD 200.7) in 2010 and reduced to 148.2 days (SD 183.1 days) in 2017. The initial sharp decline in days to the first dispensing occurred between 2012 and 2013, which coincides with pregabalin becoming available on the Pharmaceutical Benefit Scheme (PBS) in Australia (a government scheme that subsidises medicines). A lesser number of days to first gabapentinoid dispensing was associated with claimants who were part-time workers compared with full-time workers (p=0.033); those in tradespersons (p=0.048), professional (p=0.013), managers and administrator (p=0.004) occupations compared with labourers; and those in outer regional and remote areas compared with major cities (p=0.023). Other claimant characteristics were not statistically significant.

There was minimal change over time in the proportion of claimants dispensed one gabapentinoid compared with multiple dispensing (figure 1D). All gabapentinoids dispensed were for standard pack sizes, for example, 56 capsules for pregabalin, and 100 tablets for gabapentin. Pregabalin 75 mg and 150 mg capsules were the most commonly dispensed capsule strength. The mean cost of pregabalin dispensing was $A43.58 (SD $A33.72) and gabapentin $A37.18 (SD $A30.35), which represents a standard full-cost, private fee.

## Discussion

In workers with a low back pain claim, the proportion of claimants being dispensed at least one gabapentinoid during their claim increased over time despite the number of claimants being dispensed pain-related medicines decreasing. There was a significant association of a gabapentinoid dispensing with a previous opioid analgesic and anti-depressant dispensing and not claimant-level characteristics like sex, socioeconomic status or geographical location. Although the number of dispenses per claimant was stable over time, the time to first gabapentinoid dispensing became shorter over time.

Gabapentinoids play a role in managing their indicated conditions^12^ which is supported by several clinical guidelines.^24^ However, their use for other conditions can be limited and may provide low-value care. For example, in patients with sciatica, gabapentinoids may be considered as providing low-value care as they do not provide any more benefit than a placebo, only more adverse events.^17^ This is a similar case for patients with low back pain.^25 26^ Subsequently, many updated clinical guidelines now do not support gabapentinoid prescribing in these conditions.^5^ While other guidelines do not commit to a recommendation due to variations in patient preferences despite acknowledging that gabapentinoid abuse and dependence outweigh the benefits compared with placebo for patients with low back pain with or without radicular symptoms.^27^ The sequela of gabapentin being the 10th most commonly prescribed medication in 2017 in the USA^28^ and global increased gabapentinoid prescribing^14 18 19^ is the increased incidence of serious harms, such as associated deaths,^10 11 29 30^ misuse^31 32^ and non-medical use.^33–35^ In Australia, pregabalin became available on the PBS in March 2013, which saw a rapid increase in prescribing^36^ and it has continued for the proceeding years.^37^ By 2020, pregabalin had become the most supplied analgesic in Australia.^37^ Increased prescribing in Australia has also occurred to patients presenting to general practitioners with spinal pain^15^ with a similar secular trend found in our study. The increased prescribing of gabapentinoids may be associated with clinicians trying to provide a non-opioid alternative following widespread recognition of the risk of harm with opioids while still providing analgesic options to the presenting patient.^13^

There is limited literature on the extent of gabapentinoid use in workers’ compensation populations. Previous North American data has shown an increase in gabapentinoid prescribing between 2008 and 2018,^20^ similar to our study and over a similar period. However, one noticeable difference is the contrasting prescribing of the two gabapentinoids. Furthermore, the difference in drug prescribing may be related to the difference in included participants; our study was limited to low back pain-related injuries compared with all types of workers’ compensation injuries.^20^ While compensation data from Louisiana, USA, between 1998 and 2007, saw that almost all participants (98%) did not receive a gabapentinoid within the first 6 months of their claim.^38^ When a gabapentinoid was prescribed, it was associated with prolonged claim costs at 6 months.^38^

Our study is the first to report trends in gabapentinoid use in a low back pain workers compensation population. Our results are from a robust, large, externally validated database documenting claimant activity since 1996 with very minimal missing data in our data set. Our data source has the advantage over other databases as the medicine-related data is directly linked to individual claimants’ history, and hence we could determine utilisation to a specific diagnostic condition. We acknowledge there are limitations to our study. Due to the nature of the database, the data reflects only medicines available for reimbursement and does not consider medicines a claimant already had at home. Therefore, some claimants may have greater actual medicine utilisation, such as using complementary medicines. Also, our sample most likely does not include acute low back pain presentations as claimants in the database have already had 10 business days off work to be eligible for reimbursement.

There is minimal research evaluating gabapentinoid use in the workers’ compensation population. This leaves opportunities for future research. Future research could expand our research by collecting longitudinal patient-reported outcomes, such as determining any associations between gabapentinoid use in injured workers and activities of daily living, mental health, adverse events, etc. These analyses may uncover if over time workers compensation populations are at increased risk of pregabalin overdoses, a characteristic noted more frequently associated with men.^11 30^ Additionally, future analyses may investigate if co-prescribing gabapentinoids with other high-risk drugs like opioid analgesics and benzodiazepines, a triad of drugs that can have serious health consequences (eg, death, intentional or unintentional poisonings, hospitalisations), is a concern in the worker compensation population as it has been identified in the care seeing^39^ and general population.^40^

## Data Availability

No data are available. Data used in this paper are not available for distribution by the authors.
