# Supplementary material for: Secular trends in gabapentinoid dispensing by compensated workers with low back pain: a retrospective cohort study
Source: Occup Environ Med. 2024 May 23;81(5):245–51. doi: 10.1136/oemed-2023-109369 (PMC11187357; doi:10.1136/oemed-2023-109369)
Supplement: Supplementary data [file oemed-2023-109369supp001.pdf]

Appendix 1 Low back pain classification

VCode is the coding system used to classify the nature of injury/disease associated with claims.

TOOCS Parameter	***Code***	***Description***
Nature of Injury	228	Trauma to muscles and tendons, not elsewhere classified
	229	Trauma to muscles and tendons, unspecified
	239	Soft tissue injures due to trauma or unknown mechanisms
	422	Disc displacement, prolapse, degeneration or hernia
	459	Back pain, lumbago and sciatica
	488	Spinal vertebrae and intervertebral discs diseases, not elsewhere classified
	489	Spinal vertebrae and intervertebral disc diseases, unspecified
	533	Muscle / tendon strain (non-traumatic)
Bodily Location of Injury	311	Lower back
Mechanism of Injury	Any	-
Agency of Injury	Any	-

Table A1: Definition of low back pain classifications.

Appendix 2 Gabapentinoid characteristics over time

**Claimant characteristic**	**Year**								
	**2010**	**2011**	**2012**	**2013**	**2014**	**2015**	**2016**	**2017**	**Total**
Number of low back claimants (N)	2,368	2,245	2,376	2,323	2,130	2,211	2,060	1,976	17,689
Number of low back claimants with at least one gabapentinoid claims (n, % low back claimants)	188 (7.9%)	222 (9.9%)	294 (12.4%)	331 (14.2%)	334 (15.8%)	436 (19.7%)	434 (21.1%)	369 (18.7%)	2,608
Prevalence Ratio (PR) (95%CI; p-value)	Ref	1.22 (1.04, 1.44; 0.044)	1.60 (1.37, 1.86; <0.001)	1.86 (1.60, 2.15; <0.001)	2.13 (1.84, 2.46; <0.001)	2.47 (2.15, 2.84; <0.001)	2.59 (2.26, 2.98; <0.001)	2.43 (2.11, 2.81; <0.001)	-
Number of gabapentinoid dispenses per claimant among those who were dispensed a gabapentinoid (mean, SD)	7.0 (7.6)	6.0 (6.3)	6.7 (7.0)	7.8 (8.9)	7.6 (9.4)	7.7 (8.3)	8.2 (8.7)	7.0 (8.0)	**-**
Days to first gabapentinoid dispensing (mean, SD)	311.9 (200.7)	301.2 (208.7)	292.9 (206.5)	216.6 (197.0)	176.1 (195.4)	148.9 (175.7)	140.0 (176.5)	148.2 (183.1)	**-**
Median (IQR) number of gabapentinoid dispenses of those who were dispensed a gabapentinoid	4 (1, 10)	4 (1, 9)	4 (1, 10)	5 (2, 11)	4 (1, 10)	4 (1, 11)	4 (2, 13)	4 (1, 10)	**-**

Table A2: Number of gabapentinoid claimants and the number of dispensing and day to dispensing per claimant reported per year.
Abbreviations: CI = Confidence Interval; IQR = Inter-Quartile Range. SD = Standard Deviation.

Appendix 3 Episodes of gabapentinoid dispensing

Claimant characteristic	Episode of gabapentinoid dispensing[#]		
	1 episode N (%)	2 episodes N (%)	>2 episodes N (%)
**Whole sample**	1,539 (59.0)	560 (21.5)	509 (19.5)
**Year**			
2010	118 (62.8)	40 (21.3)	30 (16.0)
2011	134 (60.4)	60 (27.0)	28 (12.6)
2012	190 (64.6)	59 (20.1)	45 (15.3)
2013	186 (56.2)	75 (22.7)	70 (21.1)
2014	187 (56.0)	66 (19.8)	81 (24.3)
2015	253 (58.0)	95 (21.8)	88 (20.2)
2016	245 (56.5)	95 (21.9)	94 (21.7)
2017	226 (61.2)	70 (19.0)	73 (19.8)
**Sex**			
Female	533 (60.8)	192 (21.9)	151 (17.2)
Male	1,006 (58.1)	368 (21.2)	358 (20.7)
**Age group**			
15-24 years	70 (66.7)	20 (19.0)	15 (14.3)
25-34 years	301 (57.9)	111 (21.3)	108 (20.8)
35-44 years	447 (57.5)	181 (23.3)	149 (19.2)
45-54 years	461 (60.6)	153 (20.1)	147 (19.3)
55-64 years	243 (58.4)	91 (21.9)	82 (19.7)
65 or more years	17 (58.6)	^	^
**Employer size**			
Small	392 (58.3)	146 (21.7)	134 (19.9)
Medium	612 (57.8)	222 (21.0)	224 (21.2)
Large	426 (60.3)	163 (23.1)	117 (16.6)
Government	61 (61.6)	15 (15.2)	23 (23.2)
Missing (n = 73)	48 (65.8)	14 (19.2)	11 (15.1)
**Employment type**			
Full-time employee	1,090 (58.3)	409 (21.9)	370 (19.8)
Part-time employee	263 (63.1)	87 (20.9)	67 (16.1)
Casual	27 (57.4)	6 (12.8)	14 (29.8)
Other	159 (57.8)	58 (21.1)	58 (21.1)
**Occupation**			
Advanced clerical workers	5 (35.7)	^	^
Associate professionals	128 (59.5)	45 (20.9)	42 (19.5)
Elementary clerical, sales and service workers	72 (62.6)	29 (25.2)	14 (12.2)

Intermediate clerical, sales and service workers	185 (60.9)	70 (23.0)	49 (16.1)
Intermediate production and transport workers	327 (58.0)	117 (20.7)	120 (21.3)
Labourers and related workers	305 (55.2)	128 (23.1)	120 (21.7)
Managers and administrators	66 (68.8)	14 (14.6)	16 (16.7)
Professionals	170 (58.6)	69 (23.8)	51 (17.6)
Tradespersons and related workers	281 (61.5)	85 (18.6)	91 (19.9)
**Socioeconomic status** (Missing n = 9)			
Most advantaged	229 (64.7)	70 (19.8)	55 (15.5)
Middle three quintiles	1,030 (59.0)	367 (21.0)	350 (20.0)
Most disadvantaged	275 (55.2)	121 (24.3)	102 (20.5)
**Remoteness** (Missing n = 5)			
Major Cities	1,065 (57.8)	400 (21.7)	376 (20.4)
Inner regional	402 (62.1)	139 (21.5)	106 (16.4)
Outer regional and remote	70 (60.9)	19 (16.5)	26 (22.6)
**Medicine use**			
Dispensed an opioid analgesic(s)	1,359 (57.5)	528 (22.3)	478 (20.2)
No opioid dispensed	180 (74.1)	32 (13.2)	31 (12.8)
Dispensed an anti-depressant(s)	742 (50.6)	353 (24.1)	371 (25.3)
No anti-depressant dispensed	797 (69.8)	207 (18.1)	138 (12.1)

Table A3: Proportion and characteristics of gabapentinoid claimants per number of gabapentinoid episodes.

A new episode of gabapentinoid use was considered if there was more than 60 days between gabapentinoid service claims.

^ Values <5 are suppressed to avoid re-identification.

Episodes	***Workers***	***Episode type***			***Episode duration (days)***[3]		***Time between episodes (days)***[4]	
		Single[1]	***Multiple***[2]	***Single and Multiple***				
	N	*N (%)*	*N (%)*	*N (%)*	*Mean (SD)*	*Median (IQR)*	*Mean (SD)*	*Median (IQR)*
1 episode	1,539	714 (46.4)	825 (53.6)	- (-)	183.2 (197.9)	90 (42, 279)	- (-)	- (-)
2 episodes	560	96 (17.1)	222 (39.6)	242 (43.2)	152.5 (139.4)	102.5 (49, 219.8)	160.5 (130.9)	98 (71, 198)
>2 episodes	509	51 (10)	84 (16.5)	374 (73.5)	105.1 (93.3)	69 (41, 140.5)	116.4 (75.7)	89 (71, 133)

Table A4. N (%) of workers by the type of gabapentinoid episode (single dispense, multiple dispenses, or both), and the duration of gabapentinoid episodes and time between gabapentinoid episodes.

Abbreviations: SD = Standard Deviation; IQR = Inter-Quartile Range.

Footnotes:

1: A single gabapentinoid dispensing only.

2: More than one gabapentinoid dispensing.

3: Days between first and last gabapentinoid dispensing in a given episode, excluding single dispenses.

4: Days between episodes where the end of an episode is defined as >60 days between gabapentinoid dispensing.

In summary,

- Most workers have a single episode of gabapentinoid dispensing.
- Workers with a single episode of gabapentinoid dispensing had the longest mean episode duration (183.2 days).
- Workers with only two gabapentinoid episodes had the longest mean duration between episodes (160.5 days).

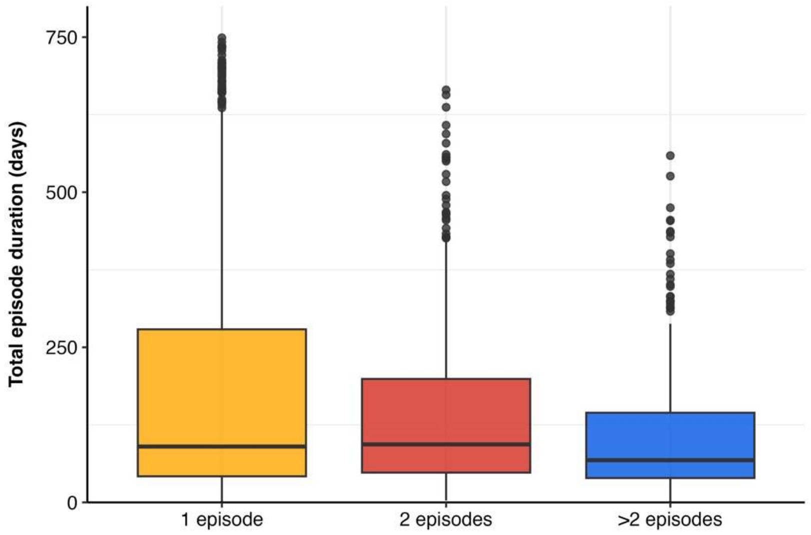

Figure A1. Gabapentinoid episode duration in workers who had one, two, or more than two episodes.

Appendix 4 Gabapentinoid dispensing relative to dispensing of other pain medicines

Tables A5 and A6 describes the proportion of low back pain (LBP) claimants who received a medicine for pain, and the pain medicine dispensing relative to their first gabapentinoid dispensing. A claimant may have received more than one class of pain medicines during their claim history, in which they are counted once in each drug class. If a claimant received multiple pain medicines within one class, they are only reported once.

Pain medicine group	***All LBP claimants (n = 17,689)***		***LBP claimants dispensed a gabapentinoid (n = 2,608)***	
	Dispensed, at any time during claimant history *N (row %)*	***Not dispensed, at any time during claimant history*** *N (row %)*	***Pain medicine dispensed, at any time during claimant history*** *N (row %)*	***No pain medicine dispensed, at any time during claimant history*** *N (row %)*
N02BF – Gabapentinoids	2,608 (14.7)	15,081 (85.3)	2,608 (100.0)	-
N02A - Opioids	5,541 (31.3)	12,148 (68.7)	2,365 (90.7)	243 (9.3)
N06A – Anti-depressants	2,476 (14.0)	15,213 (86.0)	1,466 (56.2)	1,142 (43.8)
M01A – Anti-inflammatory and antirheumatic products, non-steroids	4,223 (23.9)	13,466 (76.1)	1,809 (69.4)	799 (30.6)
M02A - Topical products for joint and muscular pain	342 (1.9)	17,347 (98.1)	141 (5.4)	2,467 (94.6)
N02BE01 - Paracetamol	2,176 (12.3)	15,513 (87.7)	1,074 (41.2)	1,534 (58.8)

Table A5. N (%) of low back pain (LBP) claimants dispensed types of medicines for pain.

Pain medicine group	***Pain medicine dispensed before gabapentinoids*** [a] *N (row %)*	***Pain medicine dispensed with or after gabapentinoids*** [b] *N (row %)*	***Total*** *N (row)*
N02A - Opioids	1,592 (67.3)	773 (32.7)	2,365
N06A – Anti-depressants	548 (37.4)	918 (62.6)	1,466
M01A – Anti-inflammatory and antirheumatic products, non-steroids	1,037 (57.3)	772 (42.7)	1,809
M02A - Topical products for joint and muscular pain	67 (47.5)	74 (52.5)	141
N02BE01 - Paracetamol	441 (41.1)	633 (58.9)	1,074

Table A6. N (%) of low back pain (LBP) claimants dispensed gabapentinoids (n = 2,608) and another medicine for pain in relation to their first gabapentinoid dispensing.

a: The first time pain medicine(s) were dispensed was before the first date of gabapentinoid dispensing.

b: The first time pain medicine(s) were dispensed was on or after the first date of gabapentinoid dispensing.

In summary

- Very few workers dispensed (or at least claimed) topical products or paracetamol.
- Most (90.7%) workers ever dispensed gabapentinoids also had opioid analgesic dispensing in this claim, of which two thirds (n = 1,592) were dispensed an opioid analgesic(s) before the gabapentinoid.
